# Diabetes-related cardiovascular and economic burden in patients hospitalized for heart failure in the US: a recent temporal trend analysis from the National Inpatient Sample

**DOI:** 10.1007/s10741-020-10012-6

**Published:** 2020-09-15

**Authors:** Menatalla Mekhaimar, Soha Dargham, Mohamed El-Shazly, Jassim Al Suwaidi, Hani Jneid, Charbel Abi Khalil

**Affiliations:** 1 Research Department, Weill Cornell Medicine—Qatar, Doha, Qatar; 2 The Michael E. DeBakey VA Medical Centre, Baylor College of Medicine, Houston, TX USA; 3 Joan and Sanford I. Weill Department of Medicine, Weill Cornell Medicine, New York, NY USA; 4 Department of Health Policy, London School of Economics, London, UK

**Keywords:** Heart failure, Diabetes mellitus, Mortality, Length of stay, Cardiovascular disease

## Abstract

We aimed to study the cardiovascular and economic burden of diabetes mellitus (DM) in patients hospitalized for heart failure (HF) in the US and to assess the recent temporal trend. Data from the National Inpatient Sample were analyzed between 2005 and 2014. The prevalence of DM increased from 40.4 to 46.5% in patients hospitalized for HF. In patients with HF and DM, mean (SD) age slightly decreased from 71 (13) to 70 (13) years, in which 47.5% were males in 2005 as compared with 52% in 2014 (*p* trend < 0.001 for both). Surprisingly, the presence of DM was associated with lower in-hospital mortality risk, even after adjustment for confounders (adjusted OR = 0.844 (95% CI [0.828–0.860]). Crude mortality gradually decreased from 2.7% in 2005 to 2.4% in 2014 but was still lower than that of non-diabetes patients’ mortality on a yearly comparison basis. Hospitalization for HF also decreased from 211 to 188/100,000 hospitalizations. However, median (IQR) LoS slightly increased from 4 (2–6) to 4 (3–7) days, so did total charges/stay that jumped from 15,704 to 26,858 USD (adjusted for inflation, *p* trend < 0.001 for both). In total, the prevalence of DM is gradually increasing in HF. However, the temporal trend shows that hospitalization and in-hospital mortality are on a descending slope at a cost of an increasing yearly expenditure and length of stay, even to a larger extent than in patient without DM.

**Electronic supplementary material:**

The online version of this article (10.1007/s10741-020-10012-6) contains supplementary material, which is available to authorized users.

## Background

Heart failure (HF) is a rising public health challenge. There are approximately 26 million worldwide suffering from HF, including more than 6.5 million people in the US [1]. HF prevalence increases gradually with age and represents a common cause of hospitalization and re-admissions, especially in the elderly [2]. It is therefore one of the leading causes of morbidity and mortality in CVD behind coronary artery disease (CAD) and stroke [3].

Diabetes mellitus (DM) and HF are often encountered together since they share many cardiovascular risk factors. Up to 40% of HF patients have DM, a prevalence that even increases more in elderly patients [4]. Several registries have already shown that the presence of DM in the general population is associated with a higher risk of developing HF on the long-term, and the presence of DM in a HF population is associated with a higher risk of cardiovascular events and rehospitalizations for HF [5, 6].

During the past decades, cardiovascular medicine has witnessed the emergence of new treatments and the implementation of primary and secondary prevention guidelines and healthcare policies, which was translated into a mortality reduction from CVD [7], in particular from CAD and stroke [8]. However, this gradual improvement in cardiovascular outcome comes at the price of an exponential increase in health expenditure in all CVD medicine specialties [9, 10]. Despite the ongoing pandemic of heart failure, temporal analysis suggests a reduction in age-specific and cause-specific mortality during the past 2 decades [11, 12]. We therefore assessed the cardiovascular and economic burden of DM in patients hospitalized for HF and examined its national trend.

## Methods

### Data source

We analyzed data from the National Inpatient Sample (NIS), which is the largest database of in-hospital patients in the US. It is part of the Healthcare Cost and Utilization Project (HCUP), which is financed by the Agency for Healthcare Research and Quality (AHRQ) [13]. Available publicly since the early 2000s till 2016, the NIS contains clinical and economic data pertinent to diagnosis and comorbidities, patients’ demographics, hospitals’ characteristics, severity and comorbidity measures, procedures, length of stay (LoS), total charges, payment sources and discharge status. There is an average of 7 million admissions collected yearly from over 1000 hospitals in 44 states, representing a stratified 20% sample of the US population, which forms almost 95% of all US admissions after weighting. Personal data are deidentified and medical acts/diagnosis are coded using the International Classification of Disease—9th edition (ICD-9) up till 2014 and ICD-10th edition afterwards.

### Diagnosis and outcomes

The primary diagnosis for this study was hospitalization for HF in patients who are 18 years of age or older (ICD-9 codes: 402.01, 402.11, 402.91, 404.01, 404.03, 404.11, 404.13, 404.91, 404.93, and all 428 sub-groups), and the secondary diagnosis was DM (ICD-9 codes: 250.0 to 250.9 with a fifth digit of 0 or 2). Patients with unknown age, gender, length of stay, in-hospital outcome, and hospital cost were excluded. Cardiovascular outcomes consisted of hospitalization for HF/100,000 adults and in-hospital mortality. Economic outcomes included length of stay (LoS) and total cost/stay.

### Statistical analysis

Baseline categorical variables and outcome measures are presented using frequency distributions, and means (standard deviations) or medians (interquartile ranges) were used for continuous variables as appropriate. We used a trend test to assess temporal changes. Comparison of HF patients with vs without DM was performed using a Student’s *t* test or a *χ*^2^ test. The total number of hospitalizations/year is weighted using a specific software to provide a nationwide estimate per the recommendation of the AHRQ [13], than presented per 100,000 population based on the yearly US population according to the US census bureau (https://www.census.gov). Briefly, patient-level discharge trend weights consisted of applying the DISCWT variable prior to 2012 and the TRENDWT variable from 2012 to 2014. Weighting results in improved national estimates, in addition to allowing for multi-year analysis of trends. In-hospital mortality is presented as crude and then stratified according to gender. Multivariable logistic regression analysis was performed to look for predictors of in-hospital mortality in patients with HF and DM. The model included age, gender, comorbidities, race, income, hospital characteristics, and the Charlson/Deyo comorbidity index; the latter being a point-based system with scores ranging from 1 to 6 with each value weighted depending on the prognostic impact of the 22 comorbidities included [14]. A Poisson regression analysis was used to estimate an annual percentage (with its 95% CI) of change in mortality and outcome. Costs were corrected for inflation using rates provided by the US bureau of labor statistics (https://data.bls.gov/cgi-bin/cpicalc.pl). A *p* value < 0.05 was considered statistically significant. Analyses were performed using SPSS (IBM, version 22.0) and STATA (version 15).

## Results

### Baseline characteristics of all patients hospitalized with heart failure

**Fig. 1:**
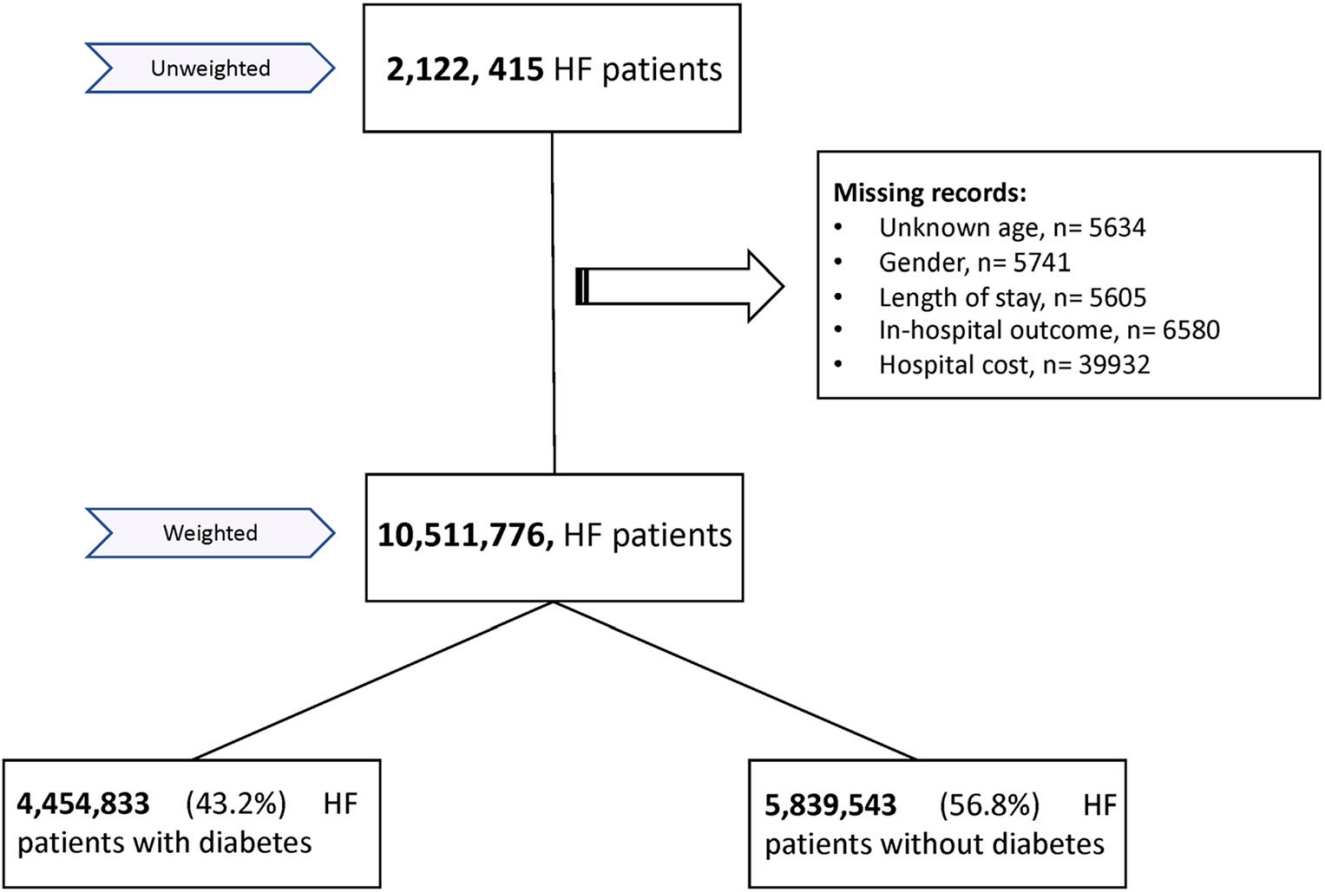
Flow chart of the analysis. HF, heart failure

A total of 2,122,415 HF patients hospitalized from 2005 to 2014 were included in our analysis after exclusion of patients with missing records (Fig. 1). After weighting, our study sample consisted of 10,511,776 HF patients: 4,454,833 (43.2%) with DM and 5,839,543 (56.8%) without DM.

Baseline characteristics of all patients with HF are shown in Supplementary Table 1. The absolute number of patients hospitalized with HF in the US gradually increased with time. Mean age (SD) decreased from 73 (14) to 72 (14) years old (*p* < 0.001). The age distribution and sex ratio significantly changed over time: The proportions of patients in the age interval 75 to 84 and > 85 gradually decreased whereas those of the age intervals < 55, 55–64, and 65–74 gradually increased (*p* trend < 0.001 for all). In 2005, there were slightly more females (51.9%), but this trend shifted to the opposite in 2014. The prevalence of DM steadily increased from 40.4 to 46.5%, which represents an absolute increase of 6.1% in a decade. The prevalence of other risk factors, such as hypertension, CAD, obesity, dyslipidemia, and smoking was also on the rise, which was translated in a temporal increase in Charlson’s score over time (*p* trend < 0.001 for all).

A similar temporal trend of age, gender ratio, and risk factors in patients with heart failure and DM and those without DM was observed. In HF patients without DM, mean age (SD) decreased from 71 (13) in 2005 to 70 (13) in 2014 (Table 1), and that of HF patients without DM moved from 74 (15) to 73 (15) (Supplementary Table 2). Cardiometabolic risk factor prevalence was on an ascending slope, so was the prevalence of CVD, such as CAD and renal failure on both groups. Interestingly, the prevalence of White Americans decreased and that of African Americans and Asians increased in diabetic HF with DM, whereas the race distribution was unchanged in HF patients without DM except of a slight increase in the prevalence of Asians.

**Table 1:** Baseline characteristics of all patients with HF from 2005 to 2014, according to the presence of Diabetes

Years	Heart failure patients with diabetes	Heart failure patients without diabetes	*p* value
	*N* = 4,454,833	*N* = 5,839,542	
Age			
Mean (SD)	71 (13)	74 (15)	< 0.001
55–64	835,952 (18.8)	680,201 (11.6)	< 0.001
65–74	1,176,474 (26.4)	961,190 (16.5)	< 0.001
75–84	1,262,978 (28.4)	1,659,077 (28.4)	< 0.001
> 84	645,476 (14.5)	1,756,911 (30.1)	< 0.001
Gender			
Male	2,221,535 (49.9)	2,883,926 (49.4)	< 0.001
Female	2,233,299 (50.1)	2,955,617 (50.6)	< 0.001
Race			
White	2,411,597 (63.1)	3,508,611 (71.6)	< 0.001
Black	823,586 (21.5)	905,400 (18.5)	< 0.001
Hispanic	386,624 (10.1)	285,625 (5.8)	< 0.001
Asian	76,382 (2)	70,737 (1.4)	< 0.001
Native American	25,370 (0.7)	23,135 (0.5)	< 0.001
Other	98,976 (2.6)	103,419 (2.1)	< 0.001
Income			
Low	1,546,020 (35.5)	1,831,534 (32)	< 0.001
Low-mid	1,162,972 (26.7)	1,517,262 (26.6)	< 0.001
High-mid	955,262 (21.9)	1,289,277 (22.6)	< 0.001
High	692,360 (15.9)	1,076,649 (18.8)	< 0.001
Insurance			
Medicare	3,288,992 (74)	4,405,484 (75.6)	< 0.001
Medicaid	389,579 (8.8)	423,920 (7.3)	< 0.001
Private insurance	556,756 (12.5)	670,562 (11.5)	< 0.001
Self-pay	122,096 (2.7)	207,756 (3.6)	< 0.001
No charge	12,877 (0.3)	20,553 (0.4)	< 0.001
Other	77,262 (1.7)	100,639 (1.7)	< 0.001
Comorbidity			
Obesity	944,309 (21.2)	544,572 (9.3)	< 0.001
Hypertension	3,150,266 (70.7)	3,513,841 (60.2)	< 0.001
Smoking	182,820 (20.3)	250,695 (21.3)	< 0.001
Dyslipidemia	399,759 (44.4)	363,226 (30.8)	< 0.001
Past medical history			
PVD	580,847 (13)	531,775 (9.1)	< 0.001
Valvular heart disease	14,116 (0.3)	20,171 (0.3)	< 0.001
Renal failure	1,955,821 (43.9)	1,790,252 (30.7)	< 0.001
CAD	474,232 (52.7)	491,700 (41.7)	< 0.001
Hospital bedsize			
Small	590,696 (14.8)	804,526 (15.6)	< 0.001
Medium	1,014,278 (25.5)	1,302,158 (25.3)	< 0.001
Large	2,379,740 (59.7)	3,043,215 (59.1)	< 0.001
Hospital location			
Rural	600,356 (15.1)	798,911 (15.5)	< 0.001
Urban	3,384,358 (84.9)	4,350,988 (84.5)	< 0.001
Hospital region			
Northeast	799,758 (20)	1,091,333 (21.1)	< 0.001
Midwest	945,667 (23.6)	1,218,489 (23.6)	< 0.001
South	1,696,082 (42.4)	2,113,830 (40.9)	< 0.001
West	562,024 (14)	750,059 (14.5)	< 0.001
Charlson score			
0	8771 (1)	274,150 (23.3)	< 0.001
1	164,377 (18.3)	439,192 (37.3)	< 0.001
2	293,598 (32.6)	284,231 (24.1)	< 0.001
≥ 3	432,676 (48.1)	181,327 (15.4)	< 0.001

PVD, peripheral vascular disease; CAD, coronary artery disease

### Temporal trend in cardiovascular outcomes

We first combined all HF patients with DM for the period of 2005 to 2014, and then compared them to those without DM for the same period. As seen in Table 2, patients with DM and HF were on average 3 years younger, more likely to belong to non-White minority groups, have a lower income, and suffered from more cardio-metabolic risk factors, such as obesity and hypertension. Cardiovascular pathologies, such as CAD and chronic renal failure, were also more prevalent. Surprisingly, the presence of DM was associated with lower in-hospital mortality risk: 111,133 deaths occurred in HF patients with DM (2.5%) versus 220,745 deaths (3.8%) in HF patients without DM, OR = 0.651 (95% CI [0.641–0.656]), *p* < 0.001. Even after multivariable adjustment on all parameters that were statistically significant between both groups, HF without DM had a lower mortality risk, adjusted OR = 0.844 (95% CI [0.828–0.860]), *p* < 0.001.

**Table 2:** Baseline characteristics and temporal trend of patients with HF and diabetes in the NIS database, between 2005 and 2014

Years	2005	2006	2007	2008	2009	2010	2011	2012	2013	2014	*p* value (trend)
Total cases	92,155	93,421	88,955	89,334	91,392	88,627	94,440	84,364	86,595	90,139	
Total cases (weighted)	451,303	457,878	440,802	437,577	463,574	443,794	454,415	421,820	432,975	450,695	
Age											
Mean (SD)	71 (13)	71 (13)	70 (13)	71 (13)	71 (13)	71 (13)	71 (13)	71 (13)	71 (13)	70 (13)	< 0.001
< 55	51,418 (11.4)	56,415 (12.3)	55,205 (12.5)	51,388 (11.7)	56,058 (12.1)	54,587 (12.3)	53,532 (11.8)	49,870 (11.8)	51,475 (11.9)	54,005 (12)	0.142
55–64	83,505 (18.5)	83,902 (18.3)	82,415 (18.7)	81,271 (18.6)	86,024 (18.6)	84,165 (19)	85,404 (18.8)	79,185 (18.8)	82,835 (19.1)	87,245 (19.4)	0.831
65–74	121,147 (26.8)	119,872 (26.2)	115,374 (26.2)	113,257 (25.9)	122,185 (26.4)	113,466 (25.6)	117,768 (25.9)	113,165 (26.8)	117,215 (27.1)	123,025 (27.3)	0.512
75–84	136,971 (30.4)	136,971 (29.7)	136,971 (29.1)	136,971 (29.4)	136,971 (28.6)	136,971 (28)	136,971 (28)	136,971 (27.1)	136,971 (26.7)	136,971 (26.5)	< 0.001
> 84	58,262 (12.9)	61,909 (13.5)	59,712 (13.5)	63,027 (14.4)	66,763 (14.4)	67,259 (15.2)	70,410 (15.5)	65,305 (15.5)	65,995 (15.2)	66,835 (14.8)	< 0.001
Gender											
Male	214,413 (47.5)	219,516 (47.9)	213,169 (48.4)	215,705 (49.3)	231,540 (49.9)	223,763 (50.4)	228,824 (50.4)	215,770 (51.2)	224,395 (51.8)	234,440 (52)	0.039
Race											
White	221,523 (67.5)	213,197 (62.1)	203,853 (61.7)	228,065 (64.4)	254,629 (63.3)	242,667 (61)	258,389 (62.4)	254,700 (63)	261,615 (63.2)	272,960 (62.9)	< 0.001
Afro-American	56,755 (17.3)	72,492 (21.1)	73,490 (22.2)	72,540 (20.5)	82,702 (20.6)	93,418 (23.5)	96,260 (23.2)	88,945 (22)	90,635 (21.9)	96,350 (22.2)	< 0.001
Hispanic	35,584 (10.8)	41,228 (12)	35,271 (10.7)	33,480 (9.5)	40,553 (10.1)	41,056 (10.3)	39,291 (9.5)	38,200 (9.5)	40,135 (9.7)	41,825 (9.6)	0.192
Asian	5252 (1.6)	6683 (1.9)	6722 (2)	7011 (2)	8172 (2)	8413 (2.1)	7043 (1.7)	8600 (2.1)	9265 (2.2)	9220 (2.1)	< 0.001
Native American	1444 (0.4)	2690 (0.8)	2654 (0.8)	2932 (0.8)	2266 (0.6)	3245 (0.8)	2534 (0.6)	2745 (0.7)	2380 (0.6)	2480 (0.6)	0.421
Other	7517 (2.3)	6953 (2)	8550 (2.6)	9884 (2.8)	13,722 (3.4)	9199 (2.3)	10,837 (2.6)	11,030 (2.7)	10,110 (2.4)	11,175 (2.6)	0.211
Income											
Low	152,120 (34.5)	161,102 (36.1)	158,946 (37)	148,923 (34.7)	154,810 (34.3)	153,439 (35.5)	158,141 (35.4)	152,220 (36.8)	149,030 (35.1)	157,290 (35.6)	0.245
Low-mid	115,937 (26.3)	115,598 (25.9)	114,949 (26.7)	120,171 (28)	122,832 (27.2)	114,916 (26.6)	112,564 (25.2)	106,315 (25.7)	114,635 (27)	125,055 (28.3)	0.737
High-mid	99,448 (22.5)	96,232 (21.5)	90,120 (21)	90,288 (21)	99,399 (22)	95,572 (22.1)	106,913 (23.9)	90,670 (21.9)	94,135 (22.2)	92,485 (20.9)	0.465
High	73,698 (16.7)	73,782 (16.5)	65,890 (15.3)	70,079 (16.3)	74,387 (16.5)	68,585 (15.9)	68,900 (15.4)	64,075 (15.5)	66,235 (15.6)	66,730 (15.1)	< 0.001
Insurance											
Medicare	339,470 (75.3)	340,195 (74.4)	322,009 (73.2)	318,351 (72.9)	337,641 (73)	321,259 (72.5)	338,868 (74.8)	316,165 (75.1)	321,895 (74.5)	333,140 (74)	0.114
Medicaid	37,973 (8.4)	39,503 (8.6)	37,675 (8.6)	35,832 (8.2)	40,500 (8.8)	41,215 (9.3)	37,925 (8.4)	36,965 (8.8)	38,105 (8.8)	43,885 (9.7)	0.439
Private insurance	54,541 (12.1)	56,644 (12.4)	58,756 (13.3)	62,486 (14.3)	60,956 (13.2)	58,183 (13.1)	55,250 (12.2)	47,290 (11.2)	49,230 (11.4)	53,420 (11.9)	0.013
Self-pay	11,800 (2.6)	11,893 (2.6)	12,216 (2.8)	10,795 (2.5)	14,342 (3.1)	13,415 (3)	11,966 (2.6)	11,730 (2.8)	13,245 (3.1)	10,695 (2.4)	0.733
No charge	1335 (0.3)	1322 (0.3)	1649 (0.4)	1252 (0.3)	1312 (0.3)	1595 (0.4)	1231 (0.3)	785 (0.2)	1185 (0.3)	1210 (0.3)	0.013
Other	5836 (1.3)	7685 (1.7)	7876 (1.8)	8197 (1.9)	7962 (1.7)	7271 (1.6)	7960 (1.8)	8015 (1.9)	8700 (2)	7760 (1.7)	0.392
Comorbidities											
Obesity	57,547 (12.8)	63,207 (13.8)	69,197 (15.7)	81,198 (18.6)	94,613 (20.4)	93,493 (21.1)	113,684 (25)	112,845 (26.8)	122,010 (28.2)	136,515 (30.3)	< 0.001
Hypertension	283,958 (62.9)	301,841 (65.9)	294,820 (66.9)	302,892 (69.2)	328,905 (70.9)	322,904 (72.8)	334,731 (73.7)	315,485 (74.8)	325,500 (75.2)	339,230 (75.3)	< 0.001
Smoking	10,493 (11.4)	12,063 (12.9)	12,743 (14.3)	13,579 (15.2)	17,912 (19.6)	18,806 (21.2)	22,427 (23.7)	21,639 (25.6)	23,917 (27.6)	29,241 (32.4)	< 0.001
Dyslipidemia	27,927 (30.3)	30,900 (33.1)	32,665 (36.7)	34,961 (39.1)	40,487 (44.3)	41,978 (47.4)	47,623 (50.4)	44,647 (52.9)	47,535 (54.9)	51,036 (56.6)	< 0.001
Past medical history											
PVD	48,707 (10.8)	49,859 (10.9)	53,552 (12.1)	56,681 (13)	60,827 (13.1)	58,766 (13.2)	64,480 (14.2)	60,420 (14.3)	62,345 (14.4)	65,210 (14.5)	< 0.001
Valvular heart disease	1255 (0.3)	1069 (0.2)	1054 (0.2)	1551 (0.4)	1620 (0.3)	1797 (0.4)	1700 (0.4)	1330 (0.3)	1295 (0.3)	1445 (0.3)	< 0.001
Chronic renal failure	105,901 (23.5)	159,894 (34.9)	177,593 (40.3)	182,052 (41.6)	209,731 (45.2)	211,265 (47.6)	227,965 (50.2)	215,795 (51.2)	224,670 (51.9)	240,955 (53.5)	< 0.001
CAD	45,401 (49.3)	45,814 (49)	44,452 (50)	45,768 (51.2)	48,792 (53.4)	47,164 (53.2)	51,859 (54.9)	47,050 (55.8)	47,876 (55.3)	50,056 (55.5)	< 0.001
Hospital bedsize											
Small	58,557 (13)	71,468 (15.7)	60,571 (13.8)	62,596 (14.3)	62,260 (13.7)	59,699 (13.6)	61,698 (13.7)	61,945 (14.7)	63,245 (14.6)	87,215 (19.4)	0.375
Medium	113,146 (25.1)	116,965 (25.6)	114,475 (26)	102,065 (23.4)	109,389 (24)	102,419 (23.3)	107,115 (23.8)	112,585 (26.7)	115,775 (26.7)	133,490 (29.6)	0.475
Large	279,599 (62)	267,985 (58.7)	264,688 (60.2)	272,328 (62.3)	284,046 (62.3)	278,084 (63.2)	281,374 (62.5)	247,290 (58.6)	253,955 (58.7)	229,990 (51)	0.007
Hospital location											
Rural	80,395 (17.8)	76,812 (16.8)	68,495 (15.6)	71,227 (16.3)	70,210 (15.4)	66,769 (15.2)	68,569 (15.2)	60,530 (14.3)	61,950 (14.3)	55,795 (12.4)	< 0.001
Urban	17.8 (370908)	16.8 (379607)	15.6 (371240)	16.3 (365761)	15.4 (385485)	15.2 (373432)	15.2 (381618)	14.3 (361290)	14.3 (371025)	12.4 (394900)	< 0.001
Hospital region											
Northeast	89,076 (19.7)	95,572 (20.9)	89,513 (20.3)	82,908 (18.9)	93,305 (20.1)	89,591 (20.2)	92,049 (20.3)	83,705 (19.8)	85,710 (19.8)	87,405 (19.4)	0.509
Midwest	108,606 (24.1)	107,624 (23.5)	109,797 (24.9)	99,843 (22.8)	114,042 (24.6)	106,040 (23.9)	107,682 (23.7)	98,000 (23.2)	98,290 (22.7)	104,350 (23.2)	0.105
South	193,290 (42.8)	192,192 (42)	180,971 (41.1)	195,077 (44.6)	190,717 (41.1)	186,242 (42)	191,788 (42.2)	178,605 (42.3)	185,890 (42.9)	194,600 (43.2)	0.064
West	60,331 (13.4)	62,490 (13.6)	60,522 (13.7)	59,749 (13.7)	65,509 (14.1)	61,922 (14)	62,896 (13.8)	61,510 (14.6)	63,085 (14.6)	64,340 (14.3)	0.336
Charlson score											
0	1631 (1.8)	1621 (1.7)	1606 (1.8)	1181 (1.3)	696 (0.8)	583 (0.7)	507 (0.5)	323 (0.4)	362 (0.4)	261 (0.3)	< 0.001
1	30,064 (32.6)	30,630 (32.8)	26,666 (30)	18,809 (21.1)	14,790 (16.2)	11,920 (13.4)	10,107 (10.7)	7844 (9.3)	7210 (8.3)	6337 (7)	< 0.001
2	33,488 (36.3)	34,445 (36.9)	32,370 (36.4)	31,523 (35.3)	30,272 (33.1)	28,697 (32.4)	28,394 (30.1)	24,658 (29.2)	24,699 (28.5)	25,052 (27.8)	< 0.001
≥ 3	26,972 (29.3)	26,725 (28.6)	28,313 (31.8)	37,821 (42.3)	45,634 (49.9)	47,427 (53.5)	55,432 (58.7)	51,539 (61.1)	54,324 (62.7)	58,489 (64.9)	< 0.001

PVD, peripheral vascular disease; CAD, coronary artery disease

We looked for predictors of mortality in those patients. As expected, increasing age is associated with higher mortality risk. For instance, patients older than 84 years have a 5-fold higher risk of dying than those 55 years of age or younger (Table 3). Females are more protected than males, and White Americans have a higher risk than all other ethnic groups. As expected, previous cardiovascular events, such as renal failure, valvular heart disease, or peripheral vascular events—but not coronary artery disease—increased significantly the risk. Interestingly, the presence of cardiometabolic risk factors, such as obesity, hypertension, dyslipidemia, and smoking, had a protective effect.

**Table 3:** Factors associated with in-hospital death in patients with HF and diabetes

Years	OR	95% CI	*p* value
Age			
< 55	1	Referent group	–
55–64	1.394	1.349 to 1.441	< 0.001
65–74	2.117	2.054 to 2.182	< 0.001
75–84	3.307	3.212 to 3.404	< 0.001
> 84	5.16	5.01 to 5.316	< 0.001
Gender			
Male	1	Referent group	–
Female	0.952	0.84 to 0.963	< 0.001
Race			
White	1	Referent group	–
Black	0.477	0.468 to 0.487	< 0.001
Hispanic	0.688	0.673 to 0.705	< 0.001
Asian	0.947	0.907 to 0.989	0.015
Native American	0.612	0.558 to 0.671	< 0.001
Comorbidity			
Obesity	0.603	0.593 to 0.613	< 0.001
HTN	0.678	0.670 to 0.686	< 0.001
Dyslipidemia	0.581	0.573 to 0.588	< 0.001
Smoking	0.596	0.586 to 0.607	< 0.001
Past medical history			
PVD	1.193	1.173 to 1.213	< 0.001
Valvular heart disease	2.839	2.657 to 3.033	< 0.001
Chronic renal failure	1.476	1.458 to 1.494	< 0.001
CAD	0.877	0.867 to 0.887	< 0.001
Hospital bedsize			
Small	1	Referent group	–
Medium	0.928	0.910 to 0.946	< 0.001
Large	0.960	0.944 to 0.977	< 0.001
Hospital location			
Rural	1	Referent group	–
Urban	0.898	0.884 to 0.913	0.001
Hospital region			
Northeast	1	Referent group	–
Midwest	0.889	0.873 to 0.905	< 0.001
South	0.520	0.496 to 0.545	< 0.001
West	0.536	0.512 to 0.561	< 0.001

PVD, peripheral vascular disease; CAD, coronary artery disease

Furthermore, we compared on a yearly basis the mortality in both groups. Unexpectedly, mortality in HF patients with DM was unexpectedly but sustainability lower from 2005 till 2014.

**Fig. 2:**
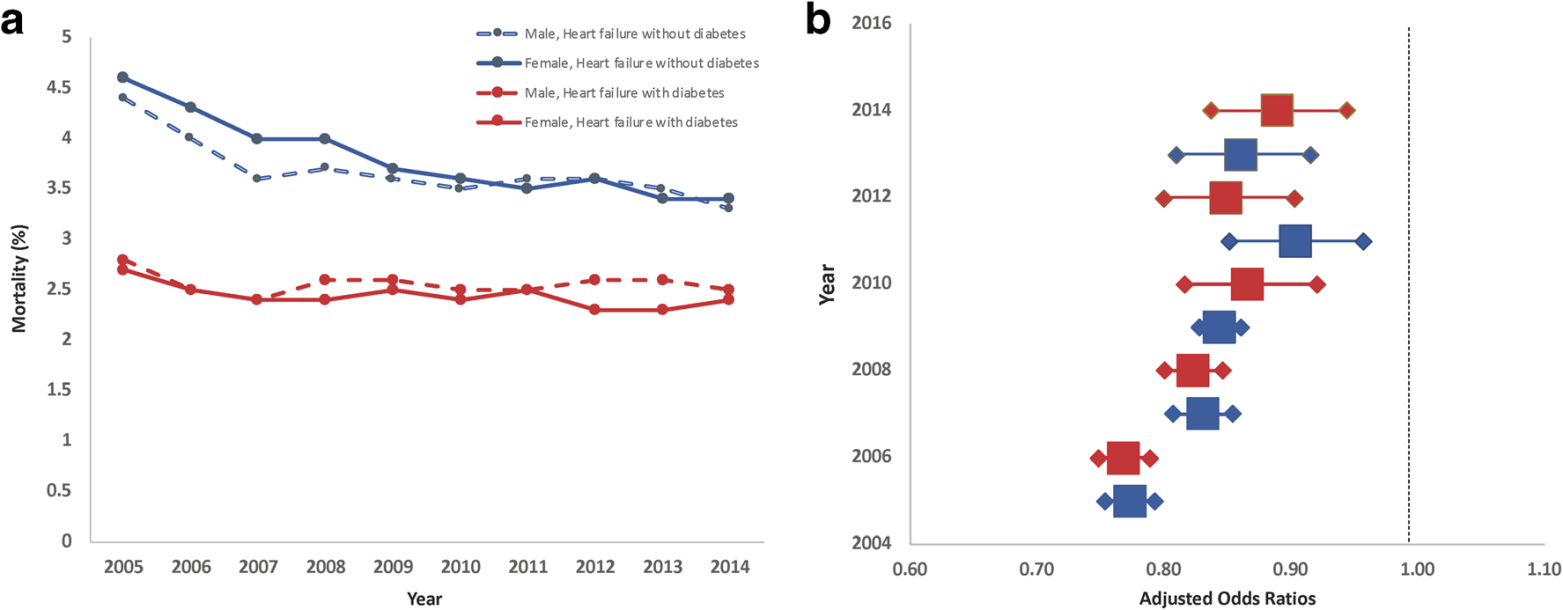
(**a**) Mortality trend in patients in patients with heart failure and diabetes (red color) and without diabetes (blue color) according to gender distribution. (**b**) Boxes represent yearly adjusted odds ratios of mortality (with its 95% CI) in patients with heart failure and diabetes

In HF patients with DM, crude mortality gradually decreased from 2.7% in 2005 to 2.4% in 2014 (Supplementary Table 3), which represents an absolute decrease of 0.3% in 10 years and an annual average decrease of 0.01% [95% CI (0.001; 0.02)] (*p* = 0.039). The reduction was observed in men (2.8% in 2005 to 2.5% in 2014) and women (2.7% in 2005 to 2.4% in 2014) (*p* trend < 0.001 for all) (Fig. 2a).

Mortality in HF patients without DM followed the same trend. Crude mortality gradually decreased from 4.5% in 2005 to 3.4% in 2014, which represents an absolute decrease of 1.1% during this same decade and an annual average decrease of 0.063% [95% CI (0.052; 0.073)] (*p* < 0.001). The reduction was observed in men (4.4% in 2005 in 2005 to 3.3% in 2014) and women (4.6% in 2005 to 3.4% in 2014) (*p* trend < 0.001 for all).

Interestingly, there was a gender effect according to the presence of DM. In HF patients without DM, women had a higher mortality risk from 2005 up till 2010 (*p* < 0.001), but no statistically significant difference in mortality is seen afterwards. In HF with DM patients, men had a higher mortality risk at all years except in 2006, 2007, and 2001 when the statistical significance was not reached, which confirmed the results of our multivariable regression analysis.

Furthermore, we performed a yearly multivariable regression analysis on all cofounding variables. Interestingly, the presence of DM was consistently associated with lower in-hospital mortality despite all adjustments from 2005 to 2014 (Fig. 2b).

**Fig. 3:**
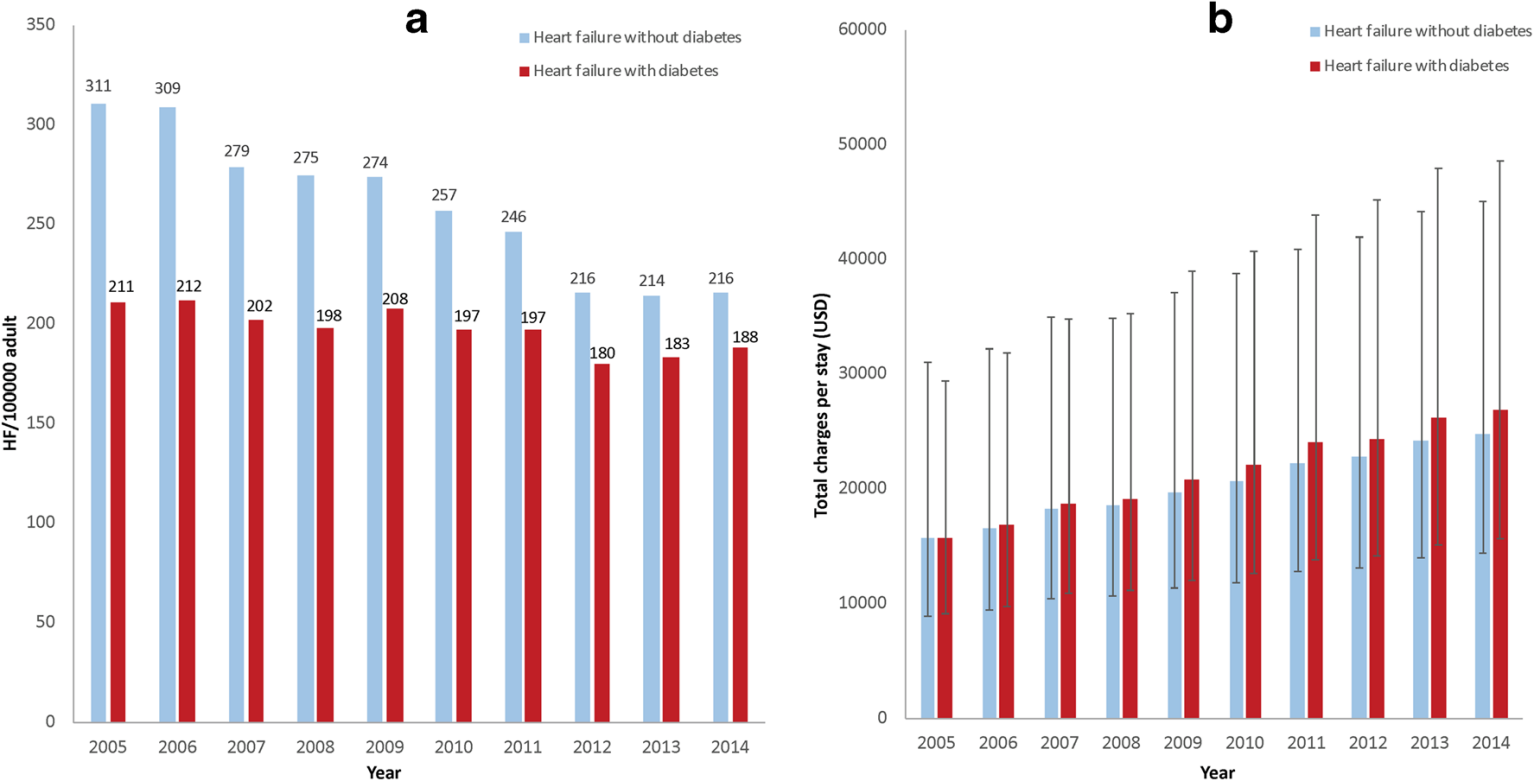
(**a**) Trend of heart failure hospitalization/100,000 US adults in patients with diabetes (red color) and without diabetes (blue color). (**b**) Temporal change in total charges/stay (median ± IQR) in heart failure patients with diabetes (red) and without diabetes (blue)

Hospitalization for HF decreased from 211/100,000 adults in 2005 to 188/100,000 adults in 2014 (*p* trend < 0.001) (Fig. 3a). A similar significant trend was also observed in patients without DM.

### Temporal trend in economic outcomes of patients with heart failure and DM

Total charges gradually increased with time: In patients with DM, charges went from 15,704 (9127–29,400) to reach 26,858 (15,638–48,590) USD/stay (adjusted for inflation, *p* trend < 0.001), which represents a mean annual increase of 5.9% (95% CI [5.4–6.5], *p* < 0.001). In HF without DM, the inflation-adjusted cost/stay also increased from 15,745 (8912–31,043) to 24,770 (14,421–45,071) USD (adjusted for inflation, *p* trend < 0.001), which represents a mean annual increase of 4.9% (95% CI [4.4–5.2], *p* < 0.001) (Fig. 3b). Of note, total charges were significantly higher in patients with DM on a yearly basis (*p* < 0.01).

The LoS was significantly lower in non-diabetic HF patients from 2005 to 2014 on a yearly basis (Supplementary Table 4). There was a slight temporal reduction in the LoS of non-diabetic HF patients from 4 (2–7) days in 2005 to 4 (2–6) days in 2014. However, the LoS slightly increased in patients with DM from 4 (2–6) to 4 (3–7) days, (*p* trend < 0.001 for both).

## Discussion

We first report in this analysis of the NIS that the prevalence of DM is gradually increasing in patients hospitalized for HF. The prevalence and incidence of DM are increasing in the general population and in individuals with previously established CVD. In a similar analysis of the NIS, Ahmed et al. reported a similar 7% absolute increase in the prevalence of DM in patients hospitalized for myocardial infarction between 2000 and 2010 [15]. Our data are also aligned with a recent temporal analysis of a large UK cohort, reporting a large increase in the prevalence of DM in HF (18% in early 2000s versus 26% in recent years) [16].

Contrary to our expectations, DM was associated with a reduced in-hospital mortality in HF despite the adjustment for confounders. Studies that reported short-term outcome in patients with HF and DM showed paradoxical results. In the OPTIMIZE-HF registry, one of the earliest and largest US performance-improvement programs in patients hospitalized with HF, in-hospital mortality did not differ according to the presence of baseline DM [17]. Similar findings were recently reported in the Scottish registry that included over 3 million participants who were followed up until 10 years [18]. Interestingly, a different larger Scottish cohort of over 110,000 HF patients reported a decreased 30-day mortality in patients with DM knowing that in-hospital mortality was not registered [19]. Concordant to our findings, a Spanish registry for over 14 years of follow-up reported a decreased in-hospital mortality in HF patients with DM [20]. Similarly, in the American “Get with the Guidelines—HF Registry,” a reduced mortality in patients hospitalized for heart failure was attributed to DM [21]. However, several other cohorts, such as the European Heart Failure registry, reported an increased risk of in-hospital death in the presence of DM [22]. Despite the existence of conflictual data in short-term outcome of patients with HF and DM, it is well known that the long-term of those patients is poor. In the Swedish National Diabetes Register, hospitalization rates in HF patients with DM were almost 50% higher as compared with the general population [23]. One year mortality and hospitalization for HF was significantly higher in HF and DM, included in the European Heart Failure registry [22].

It is not known why patients with HF and DM have a better in-hospital outcome in terms of mortality in our cohort. In our study, patients with DM have a higher prevalence of cardiometabolic parameters, such as obesity, hypertension, and renal failure. Therefore, it is highly likely that heart failure with preserved ejection fraction (HFpEF) is more prevalent than heart failure with reduced ejection fraction (HFrEF) in hospitalized, diabetic HF patients knowing that the classification into HFpEF and HFrEF was missing in the NIS before 2010. Furthermore, the composition of HF entities may have changed over time as the HFpEF’s proportion within all HF patients has recently changed in the general population and overcame that of HFrEF [24]. One of the plausible mechanisms of decreased mortality in diabetic HF patients could also be the longer LoS that probably leads to more medical acts, procedures, exploratory secondary diagnostics, and targeted treatment, which led to mortality reduction at the price of higher financial costs.

Several reports of “diabetes paradox” exist in the literature. For instance, Krinsley et al. reported that the presence of DM does not increase the risk of in-hospital death in severely ill patients admitted to the intensive care unit [25]. An obesity paradox also governs the relation between DM and mortality. Costanzo et al. showed in a large British cohort that being overweight was associated with a lower mortality risk and being obese does not increase the mortality risk as compared with average-weight individuals with DM [26]. We have recently showed that overweight, obese, and even severe obese HF patients with DM have a better short-term prognosis [27], a finding that we just confirmed in our multivariable analysis. Moreover, some of the classically harmful cardiometabolic parameters of DM, such as hypertension, dyslipidemia, and smoking, were associated with improved outcome in our study, findings that were also reported in previous NIS studies that assesses the impact of DM on other cardiovascular diseases, such as myocardial infarction [15]. One of the plausible mechanisms behind those paradoxical findings is that those patients usually receive more cardioprotective drugs that are known to decrease mortality, a factor that we could not account for in our regression model due to the absence of baseline medications in the NIS registry.

To our knowledge, we are the first to report that patients with HF and DM exert an additional cost to the healthcare system. However, our results are concordant with several reports that highlighted the financial burden of DM and its cardiovascular complications. Nichols et al. reported earlier that patients with CVD and DM are more costly than those without DM, in particular at the early course of DM [28]. Aligned with those findings, a recent systematic review that included 24 studies reported that the presence of CVD in patients with DM increased costs by 42% [29].

The temporal trend in the rate of hospitalization for HF and its associated mortality risk is concordant with current bibliography pertinent to trends and patterns of CVD and cardiovascular complications of DM in particular. In a similar analysis of the NIS, absolute risk of in-hospital mortality in patients with myocardial infraction and DM was reduced by almost 4% [15]. Burrows et al. reported a significant annual decrease of cardiovascular-related hospitalizations in patients with DM: 4.6% in patients with acute coronary syndrome, 3.6% in patients with HF, and 2% in ischemic strokes [30]. Of note, similar trends were also reported in the absence of DM.

The increasing cost of healthcare causes an enormous financial pressure on governments and funding agencies worldwide. For instance, the total cost of DM, including its comorbidities and cardiovascular complications, was estimated to be 237 billion USD in 2017, which represents a 26% increase in 5 years only [10]. According to the American Heart Association, total HF costs are expected to increase by more than twice from 2012 to 2030 [9]. As GLP-1 agonists and SGLT-2 inhibitors were only recently included in DM guidelines, we therefore anticipate a continuous reduction in DM-related mortality and hospitalization, in particular in patients with HF since the mortality reduction in those medications was mainly driven by a reduction in HF. We anticipate that newer medications and technologies in DM and HF will result in further mortality reduction given the constant evolution of medical research in cardiovascular disease. However, we also expect a continuous increase in the cost of diabetes-related complications. In fact, aging of the population which is the main driver behind the steady increase in the prevalence of CVD has been traditionally seen as the main contributor to the growing health care expenditure [31]. However, recent data indicate that advances in technologies and price growth contribute even more to healthcare spending, independently of the aging [32].

We acknowledge the presence of several limitations in this study. The NIS is an administrative database which is far from being able to generate a firm conclusion in the absence of randomization. Furthermore, a lot of the variables were not recorded. For instance, many of the mortality predictors in patients with DM and HF are missing, such as the glycemic control (HBA_1c_), LVEF, and medications. It is well known that mortality positively correlates with HBA_1C_ [33]—a marker of poor glycemic control—and LVEF and decreases with some medications, such as beta-blockers and ACE-inhibitor/angiotensin receptor blockers (ARBs) [34]. The inclusion of those variables into our regression models might have influenced the outcome. Furthermore, it was not possible to assess readmission of the same patients knowing that this outcome is one of the most important cardiovascular and economic objectives sought after in HF predictive medicine [35]. Finally, our data analysis stopped at 2014 due to the transition of ICD-9 to ICD-10 coding in 2015 in the US and the still-ongoing resulting issues in statistical decoding of the pathologies and analysis; hence, our results might not reflect accurately the trend of HF and DM in the past 5 years.

## Conclusion

The temporal trend shows that the rates of hospitalization and in-hospital mortality are on a descending slope in HF, irrespective of the presence of diabetes mellitus. However, this is counteracted by a continuous rise in the prevalence of DM and an increase in medical expenditure, notably in patients with DM who represent an additional economic burden on the growing cost of heart failure by costing more than their non-diabetic counterparts on a yearly basis.

## Electronic supplementary material

ESM 1 (DOCX 52 kb)

## Data Availability

Available upon reasonable request from the correspondent author.

## Abbreviations

CAD: coronary artery disease
HF: heart failure
HFpEF: heart failure with preserved ejection fraction
HFrEF: heart failure with reduced ejection fraction
ICD: International Classification of Diseases
LOS: length of stay
LVEF: left ventricular ejection fraction
NIS: National Inpatient Sample
