# Supplementary material for: Diabetes-related cardiovascular and economic burden in patients hospitalized for heart failure in the US: a recent temporal trend analysis from the National Inpatient Sample
Source: Heart Fail Rev. 2020 Sep 15;26(2):289–300. doi: 10.1007/s10741-020-10012-6 (PMC7895778; doi:10.1007/s10741-020-10012-6)
Supplement: Supplementary file 1 — (DOCX 52 kb) [file 10741_2020_10012_MOESM1_ESM.docx]

**Supplementary Table 1**: Baseline characteristics and temporal trend of patients with heart failure in the NIS database, between 2005 and 2014.

| **Years** | 2005 | 2006 | 2007 | 2008 | 2009 | 2010 | 2011 | 2012 | 2013 | 2014 | P value (trend) |
| --- | --- | --- | --- | --- | --- | --- | --- | --- | --- | --- | --- |
| Total cases | 228147 | 229538 | 211971 | 212749 | 211895 | 204221 | 212278 | 185961 | 187851 | 193711 |  |
| Total cases (weighted) | 1117135 | 1125210 | 1050707 | 1043328 | 1075475 | 1022455 | 1022452 | 929805 | 939254 | 968555 |  |
| Diabetes prevalence | 451303 (40.4) | 457878 (40.7) | 457878 (42) | 437577 (42) | 463574 (43.1) | 443794 (43.4) | 454415 (44.5) | 421820 (45.4) | 432975 (46.1) | 90139 (46.5) | <0.001 |
| **Age** |  |  |  |  |  |  |  |  |  |  |  |
| Mean (SD) | 73 (14) | 73 (14) | 72 (15) | 73 (14) | 73 (15) | 73 (15) | 73 (15) | 73 (14) | 72 (14) | 72 (14) | <0.001 |
| <55 | 135998 (12.2) | 148503 (13.2) | 141694 (13.5) | 131559 (12.6) | 137883 (12.8) | 134218 (13.1) | 126372 (12.4) | 116255 (12.5) | 118575 (12.6) | 125060 (12.9) | <0.001 |
| 55-64 | 154396 (13.8) | 158428 (14.1) | 152301 (14.5) | 149775 (14.4) | 156593 (14.6) | 153279 (15) | 151775 (14.8) | 139175 (15) | 146520 (15.6) | 153910 (15.9) | <0.001 |
| 65 - 74 | 235694 (21.1) | 232092 (20.6) | 216816 (20.6) | 210801 (20.2) | 221833 (20.6) | 206131 (20.2) | 207613 (20.3) | 194975 (21) | 201295 (21.4) | 210415 (21.7) | <0.001 |
| 75-84 | 347074 (31.1) | 370419 (32.9) | 337981 (32.2) | 337070 (32.3) | 338915 (31.5) | 314130 (30.7) | 310988 (30.4) | 275985 (29.7) | 270735 (28.8) | 276525 (28.6) | <0.001 |
| >84 | 243972 (21.8) | 215768 (19.2) | 201916 (19.2) | 214121 (20.5) | 220252 (20.5) | 214697 (21) | 225704 (22.1) | 203415 (21.9) | 202130 (21.5) | 202645 (20.9) | <0.001 |
| **Gender** |  |  |  |  |  |  |  |  |  |  |  |
| Male | 536841 (48.1) | 547944 (48.8) | 513477 (48.9) | 512740 (49.2) | 534426 (49.8) | 512283 (50) | 504670 (49.4) | 466140 (50.1) | 478610 (51) | 498330 (51.5) | <0.001 |
| **Race** |  |  |  |  |  |  |  |  |  |  |  |
| White | 585831 (72.7) | 559219 (67.5) | 512255 (66.1) | 577913 (69.1) | 623970 (67.8) | 603140 (66) | 626896 (67.3) | 600410 (67.5) | 605960 (67.7) | 624615 (67.3) | 0.002 |
| Afro-American | 125836 (15.6) | 158899 (19.4) | 161684 (20.9) | 155891 (19) | 176584 (19.2) | 203632 (21.8) | 197509 (21.2) | 178645 (20.1) | 179895 (20.1) | 190410 (20.5) | 0.045 |
| Hispanic | 64455 (8.0) | 73796 (9.1) | 62867 (8.4) | 59019 (7.1) | 70543 (7.7) | 70107 (7.6) | 68236 (7.3) | 65355 (7.4) | 68570 (7.7) | 69300 (7.5) | 0.065 |
| Asian | 10634 (1.3) | 13501 (1.7) | 13144 (1.8) | 13882 (1.7) | 16271 (1.8) | 16205 (1.8) | 12972 (1.4) | 16145 (1.8) | 17110 (1.9) | 17255 (1.9) | 0.007 |
| Native American | 2259 (0.3) | 5220 (0.6) | 5281 (0.7) | 5751 (0.7) | 5125 (0.6) | 6673 (0.7) | 4241 (0.4) | 5095 (0.6) | 4275 (0.5) | 4285 (0.5) | 0.172 |
| Other | 16170 (2.0) | 14462 (1.7) | 17260 (2.2) | 21386 (2.5) | 27878 (2.9) | 18344 (2) | 22219 (2.4) | 23275 (2.6) | 19755 (2.2) | 21645 (2.3) | 0.423 |
| **Income*** |  |  |  |  |  |  |  |  |  |  |  |
| Low | 354263 (32.4) | 372613 (34) | 361969 (35.2) | 336106 (33.1) | 341954 (32.6) | 337221 (33.7) | 336922 (33.1) | 316985 (34.8) | 303565 (33) | 315955 (33.3) | <0.001 |
| Low-mid | 286441 (26.2) | 283539 (25.8) | 270346 (26.6) | 290199 (28.3) | 286319 (27.4) | 262752 (26.5) | 253919 (25.2) | 231390 (25.4) | 247515 (26.9) | 267815 (28.2) | <0.001 |
| High-mid | 251201 (23.0) | 243520 (22.2) | 220359 (21.5) | 216755 (21.1) | 231924 (22.2) | 224223 (22.5) | 240357 (24.2) | 203105 (22.3) | 208425 (22.7) | 204670 (21.6) | <0.001 |
| High | 200514 (18.4) | 199473 (18) | 173208 (16.7) | 180290 (17.5) | 188299 (17.8) | 173215 (17.3) | 173030 (17.5) | 159900 (17.5) | 160575 (17.5) | 160505 (16.9) | <0.001 |
| **Insurance** |  |  |  |  |  |  |  |  |  |  |  |
| Medicare | 855511 (76.7) | 848674 (75.4) | 776639 (74) | 770869 (73.9) | 795632 (74) | 748279 (73.5) | 774052 (75.9) | 703930 (75.9) | 702934 (75) | 717955 (74.2) | <0.001 |
| Medicaid | 81243 (7.3) | 83506 (7.4) | 79357 (7.6) | 76800 (7.4) | 85401 (8) | 87402 (8.5) | 78475 (7.7) | 74510 (8) | 76015 (8.1) | 90790 (9.4) | <0.001 |
| Private Insurance | 127063 (11.4) | 132765 (11.9) | 134547 (12.8) | 140148 (13.5) | 134151 (12.6) | 128823 (12.6) | 115890 (11.4) | 98860 (10.7) | 103270 (11) | 111800 (11.6) | <0.001 |
| Self-Pay | 34239 (3.1) | 35065 (3.1) | 34853 (3.4) | 30976 (3) | 37306 (3.5) | 35491 (3.4) | 30723 (3) | 30275 (3.3) | 33010 (3.5) | 27915 (2.9) | <0.001 |
| No charge | 3489 (0.3) | 3707 (0.3) | 4409 (0.4) | 3457 (0.3) | 3378 (0.3) | 3496 (0.3) | 2992 (0.3) | 2085 (0.2) | 3635 (0.4) | 2780 (0.3) | 0.023 |
| Other | 14706 (1.3) | 19873 (1.8) | 19388 (1.9) | 19409 (1.9) | 17621 (1.7) | 16797 (1.6) | 17341 (1.7) | 17840 (1.9) | 18990 (2) | 15935 (1.6) | 0.354 |
| **Comorbidities** |  |  |  |  |  |  |  |  |  |  |  |
| Obesity | 95163 (8.5) | 103083 (9.2) | 110665 (10.5) | 130110 (12.5) | 150202 (14) | 148228 (14.5) | 175520 (17.2) | 174385 (18.8) | 188700 (20.1) | 212825 (22) | <0.001 |
| Hypertension | 628101 (56.2) | 666496 (59.2) | 634206 (60.4) | 652519 (62.5) | 699841 (65.1) | 682844 (66.8) | 697401 (68.2) | 651430 (70.1) | 662370 (70.5) | 688900 (71.1) | <0.001 |
| Smoking | 29282 (12.8) | 32762 (14.3) | 33172 (15.6) | 35068 (16.5) | 42289 (20) | 43948 (21.5) | 50866 (24) | 48858 (26.3) | 53410 (28.4) | 63860 (33) | <0.001 |
| Dyslipidemia | 55266 (24.2) | 60415 (26.3) | 63542 (30) | 68478 (32.2) | 78020 (36.8) | 80181 (39.3) | 90160 (42.5) | 84054 (45.2) | 88524 (47.1) | 94345 (48.7) | <0.001 |
| **Past Medical History** |  |  |  |  |  |  |  |  |  |  |  |
| PVD | 98407 (8.8) | 101008 (9) | 104068 (9.9) | 110633 (10.6) | 117969 (11) | 111824 (10.9) | 122333 (12) | 112685 (12.1) | 114420 (12.2) | 119275 (12.3) | <0.001 |
| Valvular heart disease | 4193 (0.4) | 3307 (0.3) | 2898 (0.3) | 3489 (0.3) | 4071 (0.4) | 3959 (0.4) | 3750 (0.4) | 2825 (0.3) | 2790 (0.3) | 3005 (0.3) | <0.001 |
| Chronic renal failure | 212384 (19) | 327049 (29.1) | 353179 (33.6) | 359746 (34.5) | 405630 (37.7) | 407854 (39.9) | 428602 (41.9) | 403010 (43.3) | 411300 (43.8) | 437320 (45.2) | <0.001 |
| CAD | 99215 (43.5) | 99404 (43.3) | 94123 (44.4) | 96281 (45.3) | 100123 (47.3) | 95540 (46.8) | 102600 (48.3) | 91545 (49.2) | 92078 (49) | 95023 (49.1) | <0.001 |
| **Hospital bedsize** |  |  |  |  |  |  |  |  |  |  |  |
| Small | 145496 (13) | 182543 (16.3) | 145631 (13.9) | 152753 (14.7) | 148790 (14.1) | 143030 (14.1) | 145101 (14.3) | 142985 (15.4) | 142074 (15.1) | 192316 (19.9) | <0.001 |
| Medium | 279960 (25.1) | 284232 (25.3) | 269267 (25.7) | 242912 (23.3) | 254916 (24.1) | 234191 (23.1) | 244538 (24.1) | 246985 (26.6) | 251505 (26.8) | 287890 (29.7) | <0.001 |
| Large | 691679 (61.9) | 655183 (58.4) | 633742 (60.4) | 646312 (62) | 654083 (61.8) | 636280 (62.8) | 623495 (61.5) | 539835 (58.1) | 545675 (58.1) | 488350 (50.4) | 0.252 |
| **Hospital location** |  |  |  |  |  |  |  |  |  |  |  |
| Rural | 194756 (17.4) | 187999 (16.8) | 168512 (16.1) | 174049 (16.7) | 166615 (15.8) | 154256 (15.2) | 156506 (15.4) | 135925 (14.6) | 134934 (14.4) | 120470 (12.4) | <0.001 |
| Urban | 922379 (82.6) | 933958 (83.2) | 880128 (83.9) | 867927 (83.3) | 891174 (84.2) | 859245 (84.8) | 856628 (84.6) | 793880 (85.4) | 804320 (85.6) | 848086 (87.6) | <0.001 |
| **Hospital region** |  |  |  |  |  |  |  |  |  |  |  |
| Northeast | 218789 (19.6) | 227677 (20.2) | 213596 (20.3) | 206152 (19.8) | 228158 (21.2) | 219994 (21.5) | 217105 (21.2) | 191750 (20.6) | 191680 (20.4) | 194980 (20.1) | <0.001 |
| Midwest | 267869 (24) | 268403 (23.9) | 262718 (25) | 245526 (23.5) | 259752 (24.2) | 239685 (23.4) | 240447 (23.5) | 211739 (22.8) | 212590 (22.6) | 223295 (23.1) | <0.001 |
| South | 473615 (42.4) | 473789 (42.1) | 424153 (40.4) | 446969 (42.8) | 435324 (40.5) | 421058 (41.2) | 423203 (41.4) | 387651 (41.7) | 392060 (41.7) | 405706 (41.9) | <0.001 |
| West | 156861 (14) | 155341 (13.8) | 150240 (14.3) | 144681 (13.9) | 152241 (14.2) | 141718 (13.9) | 141696 (13.9) | 138665 (14.9) | 142925 (15.2) | 144575 (14.9) | 0.734 |
| **Charlson Score** |  |  |  |  |  |  |  |  |  |  |  |
| 0 | 54568 (23.9) | 55411 (24.1) | 46472 (21.9) | 32357 (15.2) | 24348 (11.5) | 19948 (9.8) | 17662 (8.3) | 12235 (6.6) | 10696 (5.7) | 9224 (4.8) | <0.001 |
| 1 | 81765 (35.8) | 83080 (36.2) | 74082 (34.9) | 66480 (31.2) | 60645 (28.6) | 55426 (27.1) | 52199 (24.6) | 44213 (23.8) | 43239 (23) | 42440 (21.9) | <0.001 |
| 2 | 54322 (23.8) | 54774 (23.9) | 52835 (24.9) | 60270 (28.3) | 61655 (29.1) | 60641 (29.7) | 62803 (29.6) | 55672 (29.9) | 56427 (30) | 58430 (30.2) | <0.001 |
| ≥3 | 37492 (16.4) | 36273 (15.8) | 38582 (18.2) | 53642 (25.2) | 65247 (30.8) | 68206 (33.4) | 79614 (37.5) | 73841 (39.7) | 77489 (41.3) | 83617 (43.2) | <0.001 |

*PVD= peripheral vascular disease, CAD= coronary artery disease*

***Supplementary table 2:*** *Baseline characteristics and temporal trend of HF patients without diabetes included in the NIS database, between 2005 and 2014.*

| **Years** | 2005 | 2006 | 2007 | 2008 | 2009 | 2010 | 2011 | 2012 | 2013 | 2014 | P value (trend) |
| --- | --- | --- | --- | --- | --- | --- | --- | --- | --- | --- | --- |
| Total cases | 135992 | 136117 | 123016 | 123415 | 120503 | 115594 | 117838 | 101597 | 101256 | 103572 |  |
| Total cases (weighted) | 665832 | 667332 | 609905 | 605750 | 611901 | 578661 | 568036 | 507985 | 506280 | 517860 |  |
| **Age** |  |  |  |  |  |  |  |  |  |  |  |
| Mean (SD) | 74 (15) | 74 (15) | 74 (15) | 74 (15) | 74 (15) | 74 (16) | 75 (16) | 74 (15) | 74 (15) | 73 (15) | <0.001 |
| <55 | 84580 (12.7) | 92088 (13.8) | 86488 (14.2) | 80171 (13.2) | 81825 (13.4) | 79631 (13.8) | 72840 (12.8) | 66385 (13.1) | 67100 (13.3) | 71055 (13.7) | <0.001 |
| 55-64 | 70892 (10.6) | 74526 (11.2) | 69886 (11.5) | 68504 (11.3) | 70568 (11.5) | 69114 (11.9) | 66371 (11.7) | 59990 (11.8) | 63685 (12.6) | 66665 (12.9) | <0.001 |
| 65 - 74 | 114547 (17.2) | 112220 (16.8) | 101441 (16.6) | 97545 (16.1) | 99648 (16.3) | 92664 (16) | 89844 (15.8) | 81810 (16.1) | 84080 (16.6) | 87390 (16.9) | <0.001 |
| 75-84 | 210103 (31.6) | 202002 (30.3) | 180332 (29.6) | 178221 (29.4) | 175424 (28.7) | 160440 (27.7) | 154971 (27.3) | 135090 (26.6) | 130420 (25.8) | 132075 (25.5) | <0.001 |
| >84 | 185711 (27.9) | 186496 (27.9) | 171757 (28.2) | 181309 (29.9) | 184436 (30.1) | 176811 (30.6) | 184011 (32.4) | 164710 (32.4) | 160995 (31.8) | 160675 (31) | <0.001 |
| **Gender** |  |  |  |  |  |  |  |  |  |  |  |
| Male | 322428 (48.4) | 328429 (49.2) | 300308 (49.2) | 297035 (49) | 302886 (49.5) | 288520 (49.9) | 275846 (48.6) | 250370 (49.3) | 254215 (50.2) | 263890 (51) | <0.001 |
| **Race** |  |  |  |  |  |  |  |  |  |  |  |
| White | 364308 (76.3) | 346022 (71.8) | 308402 (69.8) | 349849 (72.9) | 369341 (71.3) | 360473 (69.3) | 368507 (71.2) | 345710 (71.3) | 344345 (71.5) | 351655 (71.3) | 0.971 |
| Afro-American | 69081 (14.5) | 86407 (17.9) | 88194 (20) | 83351 (17.4) | 93883 (18.1) | 110214 (21.2) | 101250 (19.6) | 89700 (18.5) | 89260 (18.5) | 94060 (19.1) | 0.055 |
| Hispanic | 28871 (6) | 32569 (6.8) | 27596 (6.2) | 25538 (5.3) | 29989 (5.8) | 29052 (5.6) | 28945 (5.6) | 27155 (5.6) | 28435 (5.9) | 27475 (5.6) | 0.056 |
| Asian | 5382 (1.1) | 6818  (1.4) | 6422 (1.5) | 6871  (1.4) | 8098 (1.6) | 7792 (1.5) | 5929 (1.1) | 7545 (1.6) | 7845  (1.6) | 8035  (1.6) | 0.009 |
| Native American | 1115 (0.2) | 2530  (0.5) | 2627 (0.6) | 2818  (0.6) | 2858 (0.6) | 3428 (0.7) | 1707 (0.3) | 2350 (0.5) | 1895  (0.4) | 1805  (0.4) | 0.907 |
| Other | 8653 (1.8) | 7510  (1.6) | 8710  (2) | 11503 (2.4) | 14156 (2.7) | 9145 (1.8) | 11382 (2.2) | 12245 (2.5) | 9645  (2) | 10470 (2.1) | 0.705 |
| **Income*** |  |  |  |  |  |  |  |  |  |  |  |
| Low | 202143 (31) | 211511 (32.4) | 203024 (34.1) | 187183 (31.5) | 187144 (31.3) | 183783 (32.5) | 178781 (32.1) | 164765 (33.1) | 154535 (31.2) | 158665 (31.3) | <0.001 |
| Low-mid | 170504 (26.2) | 167941 (25.7) | 155397 (26.1) | 170028 (28.6) | 163487 (27.4) | 147835 (26.2) | 141355 (25.3) | 125075 (25.1) | 132880 (26.8) | 142760 (28.1) | <0.001 |
| High-mid | 151753 (23.3) | 147288 (22.6) | 130239 (21.9) | 126467 (21.3) | 132525 (22.2) | 128651 (22.8) | 133444 (23.9) | 112435 (22.6) | 114290 (23) | 112185 (22.1) | <0.001 |
| High | 126816 (19.5) | 125691 (19.3) | 107319 (18) | 110211 (18.6) | 113912 (19.1) | 104630 (18.5) | 104130 (18.7) | 95825 (19.2) | 94340 (19) | 93775 (18.5) | <0.001 |
| **Insurance** |  |  |  |  |  |  |  |  |  |  |  |
| Medicare | 516042 (77.6) | 508479 (76.3) | 454630 (74.7) | 452518 (74.8) | 457992 (75) | 427020 (74) | 435184 (76.9) | 387765 (76.6) | 381040 (75.4) | 384815 (74.4) | <0.001 |
| Medicaid | 43270 (6.5) | 44003 (6.6) | 41682 (6.8) | 40968 (6.8) | 44900 (7.4) | 46187 (8) | 40550 (7.2) | 37545 (7.4) | 37910 (7.5) | 46905 (9.1) | 0.439 |
| Private Insurance | 72522 (10.9) | 76121 (11.4) | 75792 (12.4) | 77661 (12.8) | 73195 (12) | 70640 (12.2) | 60641 (10.7) | 51570 (10.2) | 54040 (10.7) | 58380 (11.3) | <0.001 |
| Self-Pay | 22440 (3.4) | 23172 (3.5) | 22637 (3.7) | 20181 (3.3) | 22964 (3.8) | 22076 (3.8) | 18757 (3.3) | 18545 (3.7) | 19765 (3.9) | 17220 (3.3) | <0.001 |
| No charge | 2154 (0.3) | 2386  (0.4) | 2759 (0.5) | 2205  (0.4) | 2067 (0.3) | 1902 (0.3) | 1761 (0.3) | 1300 (0.3) | 2450  (0.5) | 1570  (0.3) | 0.03 |
| Other | 8870 (1.3) | 12188 (1.8) | 11513 (1.9) | 11212 (1.9) | 9659 (1.6) | 9526 (1.6) | 9381 (1.7) | 9825 (1.9) | 10290  (2) | 8175  (1.6) | 0.32 |
| **Comorbidities** |  |  |  |  |  |  |  |  |  |  |  |
| Obesity | 37617 (5.6) | 39876  (6) | 41468 (6.8) | 48913 (8.1) | 55588 (9.1) | 54735 (9.5) | 61836 (10.9) | 61540 (12.1) | 66690 (13.2) | 76310 (14.7) | <0.001 |
| Hypertension | 344143 (51.7) | 364655 (54.6) | 339386 (55.6) | 349627 (57.7) | 370936 (60.6) | 359940 (62.2) | 362670 (63.8) | 335945 (66.1) | 336870 (66.5) | 349670 (67.5) | <0.001 |
| Smoking | 18789 (13.8) | 20699 (15.2) | 20429 (16.6) | 21489 (17.4) | 24377 (20.2) | 25142 (21.8) | 28439 (24.1) | 27219 (26.8) | 29493 (29.1) | 34619 (33.4) | <0.001 |
| Dyslipidemia | 27339 (20.1) | 29515 (21.7) | 30877 (25.1) | 33517 (27.2) | 37533 (31.1) | 38203 (33) | 42537 (36.1) | 39407 (38.8) | 40989 (40.5) | 43309 (41.8) | <0.001 |
| **Past Medical History** |  |  |  |  |  |  |  |  |  |  |  |
| PVD | 49700 (7.5) | 51149 (7.7) | 50516 (8.3) | 53952 (8.9) | 57142 (9.3) | 53058 (9.2) | 57854 (10.2) | 52265 (10.3) | 52075 (10.3) | 54065 (10.4) | <0.001 |
| Valvular heart disease | 2938 (0.4) | 2239  (0.3) | 1843 (0.3) | 1938  (0.3) | 2452 (0.4) | 2162 (0.4) | 2050 (0.4) | 1495 (0.3) | 1495  (0.3) | 1560  (0.3) | <0.001 |
| Chronic renal failure | 106482 (16) | 167155 (25) | 175586 (28.8) | 177694 (29.3) | 195899 (32) | 196588 (34) | 200637 (35.3) | 187215 (36.9) | 186630 (36.9) | 196365 (37.9) | <0.001 |
| CAD | 53814 (39.6) | 53590 (39.4) | 49671 (40.4) | 50513 (40.9) | 51331 (42.6) | 48376 (41.8) | 50741 (43.1) | 44495 (43.8) | 44202 (43.7) | 44967 (43.4) | <0.001 |
| **Hospital bedsize** |  |  |  |  |  |  |  |  |  |  |  |
| Small | 86939 (13.1) | 111075 (16.7) | 85059 (14) | 90157 (14.9) | 86530 (14.4) | 83331 (14.5) | 83403 (14.8) | 81040 (16) | 78830 (15.6) | 105100 (20.3) | 0.473 |
| Medium | 166813 (25.1) | 167266 (25.1) | 154792 (25.4) | 140847 (23.3) | 145527 (24.2) | 131772 (23) | 137423 (24.4) | 134400 (26.5) | 135730 (26.8) | 154400 (29.8) | 0.019 |
| Large | 412080 (61.9) | 387197 (58.2) | 369053 (60.6) | 373984 (61.8) | 370037 (61.5) | 358196 (62.5) | 342122 (60.8) | 292545 (57.6) | 291720 (57.6) | 258360 (49.9) | <0.001 |
| **Hospital location** |  |  |  |  |  |  |  |  |  |  |  |
| Rural | 114361 (17.2) | 111188 (16.7) | 100017 (16.4) | 102822 (17) | 96405 (16) | 87487 (15.3) | 87937 (15.6) | 75395 (14.8) | 72985 (14.4) | 64675 (12.5) | <0.001 |
| Urban | 551471 (82.8) | 554351 (83.3) | 508888 (83.6) | 502166 (83) | 505689 (84) | 485813 (84.7) | 475010 (84.4) | 432590 (85.2) | 433295 (85.6) | 453185 (87.5) | <0.001 |
| **Hospital region** |  |  |  |  |  |  |  |  |  |  |  |
| Northeast | 129713 (19.5) | 132105 (19.8) | 124083 (20.3) | 123243 (20.3) | 134853 (22) | 130403 (22.5) | 125056 (22) | 108045 (21.3) | 105970 (20.9) | 107575 (20.8) | <0.001 |
| Midwest | 159263 (23.9) | 160779 (24.1) | 152921 (25.1) | 145684 (24.1) | 145710 (23.8) | 133645 (23.1) | 132765 (23.4) | 113740 (22.4) | 114300 (22.6) | 118945 (23) | <0.001 |
| South | 280326 (42.1) | 281598 (42.2) | 243182 (39.9) | 251891 (41.6) | 244607 (40) | 234816 (40.6) | 231415 (40.7) | 209045 (41.2) | 206170 (40.7) | 211105 (40.8) | <0.001 |
| West | 96531 (14.5) | 92851 (13.9) | 89718 (14.7) | 84932 (14) | 86731 (14.2) | 79796 (13.8) | 78800 (13.9) | 77155 (15.2) | 79840 (15.8) | 80235 (15.5) | <0.001 |
| **Charlson Score** |  |  |  |  |  |  |  |  |  |  |  |
| 0 | 52937 (38.9) | 53790 (39.5) | 44866 (36.5) | 31176 (25.3) | 23652 (19.6) | 19365 (16.8) | 17155 (14.6) | 11912 (11.7) | 10334 (10.2) | 8963 (8.7) | <0.001 |
| 1 | 51701 (38) | 52450 (38.5) | 47416 (38.5) | 47671 (38.6) | 45855 (38.1) | 43506 (37.6) | 42092 (35.7) | 36369 (35.8) | 36029 (35.6) | 36103 (34.9) | <0.001 |
| 2 | 20834 (15.3) | 20329 (14.9) | 20465 (16.6) | 28747 (23.3) | 31383 (26) | 31944 (27.6) | 34409 (29.2) | 31014 (30.5) | 31728 (31.3) | 33378 (32.2) | <0.001 |
| ≥3 | 10520 (7.7) | 9548  (7) | 10269 (8.3) | 15821 (12.8) | 19613 (16.3) | 20779 (18) | 24182 (20.5) | 22302 (22) | 23165 (22.9) | 25128 (24.3) | <0.001 |

*PVD= peripheral vascular disease, CAD= coronary artery disease*

| **Years** | **2005** | **2006** | **2007** | **2008** | **2009** | **2010** | **2011** | **2012** | **2013** | **2014** |
| --- | --- | --- | --- | --- | --- | --- | --- | --- | --- | --- |
| **Mortality in heart failure patients with diabetes (%)** | 2.7 | 2.5 | 2.4 | 2.5 | 2.5 | 2.5 | 2.5 | 2.5 | 2.5 | 2.4 |
| **Mortality in heart failure patients without diabetes (%)** | 4.5 | 4.2 | 3.8 | 3.9 | 3.7 | 3.5 | 3.6 | 3.6 | 3.5 | 3.4 |
| **Odds Ratios**  **(95% CI)** | 0.59  (0.57-0.60) | 0.58  (0.573 - 0.59) | 0.62  (0.60 - 0.63) | 0.63  (0.62 - 0.65) | 0.67  (0.62 - 0.69) | 0.68  (0.67 - 0.70) | 0.69  (0.68 - 0.71) | 0.67  (0.65 - 0.69) | 0.71  (0.69 - 0.72) | 0.71  (0.70 - 0.73) |
| **P value** | <0.001 | <0.001 | <0.001 | <0.001 | <0.001 | <0.001 | <0.001 | <0.001 | <0.001 | <0.001 |

***Supplementary table 3:*** *In-hospital mortality in heart failure patients according to the presence of diabetes, from 2005 to 2014.*

***Supplementary table 3:*** *Length of stay in heart failure patients according to the presence of diabetes, from 2005 to 2014.*

| **Years** | | **2005** | | **2006** | | **2007** | | **2008** | | **2009** | | **2010** | | **2011** | | **2012** | | **2013** | | **2014** |  |
| --- | --- | --- | --- | --- | --- | --- | --- | --- | --- | --- | --- | --- | --- | --- | --- | --- | --- | --- | --- | --- | --- |
| LoS in HF patients with diabetes, median (IQR) | 4 (2 - 6) | | 4 (2 - 6) | | 4 (3 - 6) | | 4 (3 - 7) | | 4 (2 - 6) | | 4 (2 - 6) | | 4 (2 - 6) | | 4 (3 - 6) | | 4 (3 - 7) | | 4 (3 - 7) | | |
| LoS in HF patients without diabetes, median (IQR) | 4 (2 - 7) | | 4 (2 - 6) | | 4 (2 - 6) | | 4 (2 - 6) | | 4 (2 - 6) | | 4 (2 - 6) | | 4 (2 - 6) | | 4 (2 - 6) | | 4 (2 - 6) | | 4 (2 - 6) | | |
| **P value** | <0.001 | | <0.001 | | <0.001 | | <0.001 | | <0.001 | | <0.001 | | <0.001 | | <0.001 | | <0.001 | | <0.001 | | |

*LOS= length of stay, HF= heart failure*
